# Nonlinear Thermal/Mechanical Buckling of Orthotropic Annular/Circular Nanoplate with the Nonlocal Strain Gradient Model

**DOI:** 10.3390/mi14091790

**Published:** 2023-09-19

**Authors:** Mostafa Sadeghian, Arvydas Palevicius, Giedrius Janusas

**Affiliations:** Faculty of Mechanical Engineering and Design, Kaunas University of Technology, Studentu 56, 51424 Kaunas, Lithuania; arvydas.palevicius@ktu.lt (A.P.);

**Keywords:** nonlinear, thermal/mechanical buckling, nonlocal strain gradient theory, annular/circular nanoplate, single-layer/bilayer, HSDT, DQM

## Abstract

This article presents the nonlinear investigation of the thermal and mechanical buckling of orthotropic annular/circular single-layer/bilayer nanoplate with the Pasternak and Winkler elastic foundations based on the nonlocal strain gradient theory. The stability equations of the graphene plate are derived using higher-order shear deformation theory (HSDT) and first-order shear deformation theory (FSDT) considering nonlinear von Karman strains. Furthermore, this paper analyses the nonlinear thermal and mechanical buckling of the orthotropic bilayer annular/circular nanoplate. HSDT provides an appropriate distribution for shear stress in the thickness direction, removes the limitation of the FSDT, and provides proper precision without using a shear correction coefficient. To solve the stability equations, the differential quadratic method (DQM) is employed. Additionally, for validation, the results are checked with available papers. The effects of strain gradient coefficient, nonlocal parameter, boundary conditions, elastic foundations, and geometric dimensions are studied on the results of the nondimensional buckling loads. Finally, an equation is proposed in which the thermal buckling results can be obtained from mechanical results (or vice versa).

## 1. Introduction

Nanoplates including graphene sheets can be named as one category of eminent materials for the next generation of nanodevices. Graphene sheets can be utilized in the manufacture of many nanodevices like sensors and memory devices [1] and there are other applications such as nanosheet resonators [2], mass sensors [3], and gas sensors [4]. Because performing highly accurate experiments at the nanoscale is very demanding and high-cost, some techniques such as continuum-based modelling and molecular dynamics (MD) have been employed recently that have the potential to consider the atomic size and length effects. MD modelling of nanoscale structures needs time-consuming procedures and complicated computations. Continuum modelling of nanostructures is computationally less expensive than MD modelling; therefore, continuum theories have been formulated and employed for the investigation of the mechanical properties of nanoscale structures. In other words, continuum modelling of nanostructures can assist in understanding the results of experimental measurements (or MD modelling) more properly, because it can decrease the volume and computational time. In order to make computations easier, continuum mechanics can be employed as an appropriate option for theoretical investigations. The atomic length scales and interatomic force coefficients can be incorporated into the constitutive equations. In the classical continuum model, the influences of size in nanostructures are not regraded [5,6].

Experimental investigations have proved that there are size-dependent effects on the mechanical properties at the nanostructures [7]. Therefore, modified continuum theories are required to consider small-scale influences and maintain the ease and computational efficiency of continuum theories. Consequently, new theories have been suggested to take into account nanoscale effects in nanostructures. Various nonclassical continuous models have been presented considering the material length scale coefficient, and multiple modifications to classical elasticity theories including Eringen’s nonlocal elasticity theory [8], the strain gradient theory [9], and the nonlocal strain gradient theory [10] have been proposed.

Narendar [11] perused the buckling of rectangular nanoplates on the refined basis of the two-variable plate theory considering nonlocal scale through the Navier technique. He observed that by increasing the size of the square nanoplate, the buckling load ratio decreases significantly. Jamalpoor and his colleagues [12] studied the vibration and buckling of nanoplates exposed to electric as well as magnetic potentials based on the nonlocal plate theory. They noticed that the buckling load increased by increasing the external magnetic potential. Li and Hu [13] used nonlocal continuum theory to study the buckling, vibration, and bending of composite nanobeams. They concluded that the critical buckling load can be increased by reducing the nonlocal coefficient when the nonlocal coefficient is larger than the material characteristic coefficient. Shaban and Alibeigloo [14] investigated the bending and vibration analysis of carbon nanotubes on elastic foundations using the nonlocal three-dimensional theory of elasticity. They observed that the static and vibrational behaviors of carbon nanotubes are significantly affected by elastic foundations. Rokni et al. [15] studied the free vibration of circular/annular plates considering variable thickness with various boundary conditions using three-dimensional elasticity. Shaban and Mazaheri [16] analyzed the electrostatic behavior of microsandwich panels with the functionally graded core using the nonlocal three-dimensional theory of elasticity. They observed that increasing the nonlocal coefficient led to an increase in the displacements. The authors of Ref. [17] perused the free vibration of functionally graded plates on the elastic foundation with elastically restrained edges using FSDT. In their paper, they studied the influences of coefficients including thickness-to-radius, material distribution, various combinations of constraints at edges on the frequency, foundation stiffness coefficients, mode shape, and modal stress.

It is noticed that in the former analyses, the small-scale effect in sort of the stress nonlocality led to a softening–stiffness effect, while the strain gradient size dependency resulted in a hardening–stiffness effect. Consequently, various nonclassical continuum theories of elasticity have been presented to prognosticate various size dependencies in the mechanical features of nanostructures. The theory of nonlocal strain gradients is one of the prominent models for analyzing size-dependent mechanical features. Contrary to classical elasticity theory, the nonlocal strain gradient theory can consider the influences of stiffness–hardening and stiffness–softening by assuming the strain and stress gradient. Therefore, Lim and his colleagues [10] studied a new size-dependent elasticity model named nonlocal strain gradient theory considered both softening and stiffening effects to illustrate the size dependency more accurately.

Some analyses have been carried out with respect to the nonlocal strain gradient theory [18]. For example, the authors of Ref. [19] investigated the buckling of the nanocrystal shell with the axial loads assuming thermal, magnetic, and electrical conditions with the nonlocal strain gradient theory. They deduced that the nonlocal parameter affects the critical buckling load more notably for nonlocal parameters ranging from 2 to 20 nm. Tanzadeh and Amoushahi [20] perused the buckling of rectangular nanoplates subjected to various uniaxial in-plane loads using the nonlocal strain gradient theory with the higher-order finite strip technique. They concluded that increasing the strain gradient coefficient resulted in an increase in buckling load. Cuong-Le et al. [21] studied the bending, buckling, and vibration of sandwich nanoplates based on the theory of nonlocal strain gradients. They noticed that the nonlocal and strain gradient coefficients result in stiffness reduction and stiffness hardening cases. Therefore, both of these coefficients play a significant effect in the buckling of sandwich nanoplate. Wang and his colleagues [22] perused the buckling of functionally graded nanotubes based on the nonlocal strain gradient theory with high-order theory via the generalized differential quadrature technique. Wang et al. [23] analyzed the buckling of porous functionally graded porous nanobeams considering hybrid effects using the nonlocal strain gradient principle with the aid of the generalized differential quadrature technique. Esen and Özmen [24] studied thermal vibration and buckling functionally graded porous rectangular nanoplates considering magnet electroelastic effects based on the nonlocal strain gradient theory and FDST. They revealed that the temperature increase influences the buckling characteristic of the nanoplate depending on the proportions of the constituents in the nanoplate. Tang and Qing [25] examined the buckling and vibration of functionally graded beams based on the theory of nonlocal strain gradient and using the Laplace transform method. They scrutinized that the effects of length-scale coefficients on the vibration frequencies and buckling loads increase with the rise in vibration and buckling order. Al-Furjan and his colleagues [26] conducted research on the dynamic buckling of carbon nanocones with the nonlocal strain gradient theory and FSDT. They demonstrated that by increasing the strain gradient coefficient, the dynamic instability region occurred at high frequencies. Magorzata Chwa and Muc [27] utilized the nonlocal strain theory and considered higher-order shear deformation theories to analyze buckling and free vibrations of rectangular nanoplates via the Rayleigh–Ritz method. Using nonlocal strain gradient elasticity theory and third-order shear deformation plate theory, Fan et al. [28] studied buckling and postbuckling porous functionally graded nanoplates. They noticed that by increasing the material characteristic gradient index, both nonlocal and strain gradient size influences are noticeable. Sadeghian et al. [29] studied the investigation of large deflection of the annular/circular nanoplate on the basis of nonlocal strain gradient theory. They concluded that both strain gradient and nonlocal coefficients have noticeable effects on decreasing or increasing the deflection of the nanoplate.

One of the important issues in evaluating the stability of various structures is the buckling analysis, which has drawn the attention of many researchers to perform research on it. The term buckling refers to the loss of stability, or in other words, when the structure changes from stable equilibrium to unstable equilibrium, the structure undergoes buckling. In fact, the behavior of the structure under load in which with a small increase in load a disproportionate increase in displacement occurs in the structure is called buckling, and the amount of force for which the structure buckles is called critical buckling load [30]. There are many papers that consider buckling analysis. For example, Zghal et al. [31] scrutinized the postbuckling of functionally graded nanotubes considering various mechanical loadings. In their article, they discussed the influences of the carbon-nanotubes (CNT) volume fractions, CNT distributions, gradient indexes, and boundary conditions. In another paper, Zghal and Dammak [32] studied the buckling of functionally graded structures subjected to various compression loads. They noticed that the evenly porous FGM structures have the minimum buckling load while the uneven ones possess the maximum one. Trabelsi et al. [33] studied thermal buckling of functionally graded shells. They demonstrated that the flexural rigidity of the FG cone exposed to thermal loads can be improved with the variation of the power-law parameter. Mehr et al. [34] studied functionally graded CNT-reinforced composite shells with thermal buckling. They noticed that increasing aspect ratio and CNT volume fraction cause higher critical buckling temperature. Van Do and Lee [35] investigated the thermal buckling of FGM plates based on the quasi-3D higher-order shear deformation theory. They noticed that the temperature distribution on the plate noticeably influences the thermal buckling responses of FGM plates.

DQM is one of the numerical methods that has been employed by many authors [36,37]. The initial purpose of this procedure is to employ Lagrange interpolation polynomials to field constants and to solve the equations at discrete grid nodes. Higher accuracies are gained by considering more grid nodes. With the aid of DQM, Han et al. [38] studied one-electrode microresonators on the basis of a generalized 1DOF principle. Duryodhana et al. [37] employed DQM to study the buckling (and free vibrations) of foamed composites. By means of DQM, Ren et al. [39] investigated the thermal and mechanical buckling behavior of heated rectangular plates considering the characteristics of the temperature-dependent material.

FSDT accounts for the shear deformation influences by the way of linear variation of in-plane displacements through the thickness. Because the FSDT violates the conditions of zero transverse shear stresses on the top and bottom surfaces, a shear correction factor (which can depend on many parameters) is needed to compensate for the error caused by a constant shear strain assumption through the thickness. The HSDTs account for the shear deformation influences and satisfy the zero transverse shear stresses on the top and bottom surfaces; therefore, a shear correction factor is not needed. HSDTs are modelled based on the assumption of higher-order variations of in-plane displacements or both in-plane and transverse displacements through the thickness. Some of HSDTs are computational costs because with each additional power of the thickness coordinate, an additional unknown can be added to the model [40]. Also, there is quite the difference between FSDT and HSDT behavior for thick plates, but for thin plates both of the theories predict approximately similar result [41].

Until now, the nonlocal strain gradient theory with HSDT and FSDT has not been carried out for studying the nonlinear thermal as well as mechanical buckling of the orthotropic annular/circular nanoplate via DQM. Furthermore, the buckling analysis of a bilayer annular/circular nanoplate with elastic foundations is also studied. The effects of the nonlocal parameter, strain gradient coefficient, boundary conditions, geometric dimensions, and elastic foundations on the results of the nondimensional buckling loads are investigated. From the results, it can be concluded that the effect of the nonlocal parameter on the buckling of the circular/annular nanoplate is more significant than the types of boundary conditions or elastic foundations. Also, it is noticed that a bilayer nanoplate cannot be assumed as an equivalent single-layer nanoplate (with the same thickness as the bilayer nanoplate) to gain the results of the buckling of the nanoplate. The results of the current article can be a benefit for the design as well as improvement of nanostructured devices, including circular gate transistors, microswitches, and so on.

## 2. Theory and Formulation

**Figure 1:**
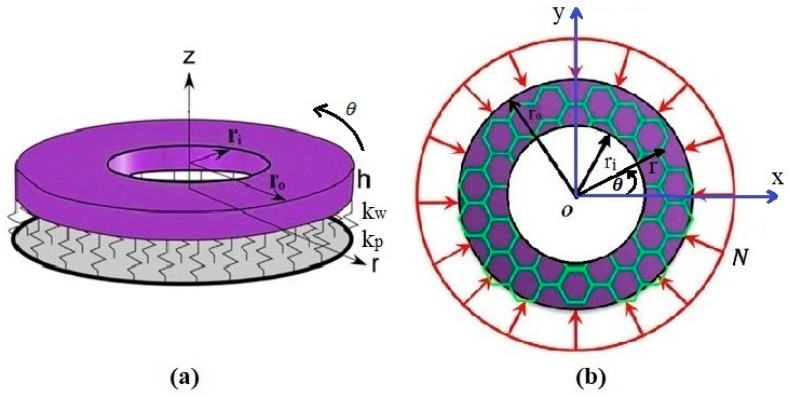
(**a**) The annular nanoplate with Pasternak–Winkler elastic foundation; (**b**) the graphene plate with uniform buckling loads.

Figure 1a,b demonstrate an annular graphene plate with inner radius $r_{i}$, outer radius $r_{o},$ and constant thickness h on the Winkler–Pasternak elastic foundation (in which $k_{p}$ and $k_{w}$ define the Pasternak and Winkler elastic foundations) and under uniform extended buckling loads.

Taking into account the HSDT, the displacement field can be written in the *r*, *θ*, and *z* axes clarified by *U*, *V*, and *W*, respectively.

(1) $$\begin{array}{l} U(r,\theta ,z)=u_{0}(r)-z\frac{dw_{0}(r)}{dr}+g(z)\phi (r)\\ V(r,\theta ,z)=0\\ W(r,\theta ,z)=w_{0}(r) \end{array}$$

where $u_{0}$ and $w_{0}$ define the displacements of the midplane on the *r* and *z* axes, respectively. Furthermore, ϕ(r) is the rotation component around the *θ* axis. Also, the function *g*(*z*) can be mentioned as:

(2) $$g(z)=f(z)+zy^{*}.$$

*f* (*z*) and y* can be assumed as various functions utilized in different papers which are listed in Table 1 (for instance, the Ambartsumian [42] model can be considered as $\underset{f(z)}{\underset{︸}{-\frac{1}{6}z^{3}}}+\underset{y^{*}}{\underset{︸}{\frac{h^{2}}{8}}}z)$.

The nonlinear strains, considering von Karman’s presumptions, can be written as Equations (3)–(7):

(3) $$\varepsilon_{r}=\frac{dU}{dr}+\frac{1}{2}{\left(\frac{dW}{dr}\right)}^{2}=\frac{du_{0}}{dr}-z\frac{d^{2}w_{0}}{dr^{2}}+g\left(z\right)\frac{d\phi }{dr}+\frac{1}{2}{\left(\frac{dw_{0}}{dr}\right)}^{2}\;,$$

(4) $$\varepsilon_{\theta }=\frac{U}{r}=\frac{1}{r}\left(u_{0}-z\frac{dw_{0}}{dr}+g\left(z\right)\phi \right),$$

(5) $$\gamma_{r\theta }=0,$$

(6) $$\gamma_{rz}=\frac{dW}{dr}+\frac{dU}{dz}=\phi \frac{dg\left(z\right)}{dz},$$

(7) $$\gamma_{\theta z}=0.$$

It is noted since the symmetric assumption is considered, Equations (5) and (7) are equal to zero.

The nonlocal form (*NL*) of the force and momentum resultants can be clarified as:

(8) $${\left\{N_{r},N_{\theta },Q_{r}\right\}}^{NL}=\int_{-\frac{h}{2}}^{\frac{h}{2}}{\left\{\sigma_{r},\sigma_{\theta },\sigma_{rz}\right\}}^{NL}dz\;,$$

(9) $${\left\{M_{r},M_{\theta }\right\}}^{NL}=\int_{-\frac{h}{2}}^{\frac{h}{2}}{\left\{\sigma_{r},\sigma_{\theta }\right\}}^{NL}zdz\;,$$

(10) $${\left\{R_{r},R_{\theta }\right\}}^{NL}=\int_{-\frac{h}{2}}^{\frac{h}{2}}{\left\{\sigma_{r},\sigma_{\theta }\right\}}^{NL}f\left(z\right)dz\;,$$

(11) $$R_{rz}^{NL}=\int_{-\frac{h}{2}}^{\frac{h}{2}}\sigma_{rz}^{NL}f^{\prime }\left(z\right)dz\;.$$

The potential energy of the system is the sum of the strain energy created due to the work of internal forces as well as the potential energy created by external forces.

(12) Π=U+Ω

Π, *U*, and Ω are described as the potential energy of the entire system, the strain energy of the structure, and the potential energy of external forces, respectively. According to the concept of minimum potential energy, for a structure in equilibrium, the variation in the potential energy is equal to zero.

(13) δΠ=δU+δΩ=0

By zeroing δΠ, the coefficients of $\delta u_{0},\delta w_{0}$, and δϕ must be equal to zero. The Euler-Lagrange equations (which are in non-local form, so they are marked using superscript *NL)* are obtained as

(14) $$\delta u_{0}:N_{r}^{NL}+r\frac{dN_{r}^{NL}}{dr}-N_{\theta }^{NL}=0,$$

(15) $$\delta \phi :y^{*}\left(r\frac{dM_{r}^{NL}}{dr}+M_{r}^{NL}-M_{\theta }^{NL}-rQ_{r}^{NL}\right)+R_{r}^{NL}+r\frac{dR_{r}^{NL}}{dr}-R_{\theta }^{NL}-rR_{rz}^{NL}=0,$$

(16) $$\begin{array}{l} \delta w_{0}:r\frac{d^{2}M_{r}^{NL}}{dr^{2}}+2\frac{dM_{r}^{NL}}{dr}-\frac{dM_{\theta }^{NL}}{dr}+\frac{dw_{0}}{dr}\left(N_{r}^{NL}+\frac{dN_{r}^{NL}}{dr}r\right)+rN_{r}^{NL}\frac{d^{2}w_{0}}{dr^{2}}+\\ \left(-k_{w}w_{0}+k_{p}\nabla^{2}w_{0}\right)r=0 \end{array}.$$

Also, Equation (16) can be rewritten as

(17) $$\begin{array}{l} \delta w_{0}:r\frac{d^{2}M_{r}^{NL}}{dr^{2}}+2\frac{dM_{r}^{NL}}{dr}-\frac{dM_{\theta }^{NL}}{dr}+\frac{dw_{0}}{dr}N_{\theta }^{NL}+rN_{r}^{NL}\frac{d^{2}w_{0}}{dr^{2}}\\ +\left(-k_{w}w_{0}+k_{p}\nabla^{2}w_{0}\right)r=0 \end{array}.$$

The nonlocal strain gradient theory (assumed as a combination of both the strain gradient theory and the nonlocal stress field) was suggested by Lim et al. [10]:

(18) $$(1-\mu^{2}\nabla^{2})\sigma_{ij}=C_{ijkl}(1-l^{2}\nabla^{2})\varepsilon_{kl},\nabla^{2}=\frac{d^{2}}{dr^{2}}+\frac{1}{r}\frac{d}{dr}$$

in which $C_{ijkl},l$, and μ define elastic, strain gradient (internal material length scale), and nonlocal parameters, respectively. In addition, the constitutive stress–strain equation in the very small scale is defined as [49]

(19) $$\begin{array}{l} (1-\mu^{2}\nabla^{2})\left[\begin{array}{l} \sigma_{r}\\ \sigma_{\theta }\\ \sigma_{rz} \end{array}\right]=(1-l^{2}\nabla^{2})\left[\begin{array}{ccc} Q_{11} & Q_{12} & 0\\ Q_{12} & Q_{22} & 0\\ 0 & 0 & G_{13} \end{array}\right]\left[\begin{array}{l} \varepsilon_{r}\\ \varepsilon_{\theta }\\ \gamma_{rz} \end{array}\right],\;\\ \left\{\begin{array}{l} Q_{11}=\frac{E_{1}}{1-\nu_{12}\nu_{21}}\;,\;Q_{22}=\frac{E_{2}}{1-\nu_{12}\nu_{21}}\\ Q_{12}=\frac{\nu_{12}E_{2}}{1-\nu_{12}\nu_{21}}\;,\;G_{13}=\frac{E_{1}}{2(1+v_{12})} \end{array}\right. \end{array}.$$

It should be mentioned in Equation (19), $E_{1}$ and $E_{2}$ indicate the Young modulus along the 1 and 2 directions. Additionally, $v_{12}$ and $v_{21}$ are Poisson ratios in those directions. Also, $G_{13}$ signifies the shear modulus.

The nonlocal form can be mentioned as

(20) $$\left(1-\mu \nabla^{2}\right){\left\{N_{r},N_{\theta },Q_{r}\right\}}^{NL}=\int_{-\frac{h}{2}}^{\frac{h}{2}}\left(1-\mu \nabla^{2}\right){\left\{\sigma_{r},\sigma_{\theta },\sigma_{rz}\right\}}^{NL}dz.$$

The force and moment resultants in the local form can be written as

(21) $${\left\{N_{r},N_{\theta },Q_{r}\right\}}^{L}=\int_{-\frac{h}{2}}^{\frac{h}{2}}{\left\{\sigma_{r},\sigma_{\theta },\sigma_{rz}\right\}}^{L}dz\;,$$

(22) $${\left\{M_{r},M_{\theta }\right\}}^{L}=\int_{-\frac{h}{2}}^{\frac{h}{2}}{\left\{\sigma_{r},\sigma_{\theta }\right\}}^{L}zdz\;,$$

(23) $${\left\{R_{r},R_{\theta }\right\}}^{L}=\int_{-\frac{h}{2}}^{\frac{h}{2}}{\left\{\sigma_{r},\sigma_{\theta }\right\}}^{L}f\left(z\right)dz\;,$$

(24) $$R_{rz}{}^{L}=\int_{-\frac{h}{2}}^{\frac{h}{2}}\sigma_{rz}{}^{L}f^{\prime }\left(z\right)dz\;.$$

Moreover, the resultants in terms of displacements are gained as follows:

(25) $$\begin{array}{l} N_{r}^{L}=(1-l^{2}\nabla^{2})\{\frac{1}{1-\nu_{12}\nu_{21}}\left(E_{1}h\right.\left(\frac{du_{0}}{dr}+\frac{1}{2}{\left(\frac{dw_{0}}{dr}\right)}^{2}\right)+\nu_{12}E_{2}h\frac{1}{r}u_{0}\\ +\left(E_{1}\frac{d\phi }{dr}+\nu_{12}E_{2}\frac{1}{r}\phi \right)\int_{-\frac{h}{2}}^{\frac{h}{2}}\left.f\left(z\right)dz\right)\} \end{array},$$

(26) $$\begin{array}{l} N_{\theta }^{L}=(1-l^{2}\nabla^{2})\{\frac{1}{1-\nu_{12}\nu_{21}}\left(\nu_{12}E_{2}h\right.\left(\frac{du_{0}}{dr}+\frac{1}{2}{\left(\frac{dw_{0}}{dr}\right)}^{2}\right)+E_{2}h\frac{1}{r}u_{0}+\\ \left(\nu_{12}E_{2}\frac{d\phi }{dr}+E_{2}\frac{1}{r}\phi \right)\int_{-\frac{h}{2}}^{\frac{h}{2}}\left.f\left(z\right)dz\right)\} \end{array},$$

(27) $$\begin{array}{l} M_{r}^{L}=(1-l^{2}\nabla^{2})\{\frac{1}{1-\nu_{12}\nu_{21}}\left(E_{1}\right.\frac{h^{3}}{12}\left(-\frac{d^{2}w_{0}}{dr^{2}}+y^{*}\frac{d\phi }{dr}\right)+\\ \nu_{12}E_{2}\frac{h^{3}}{12}\left(-\frac{1}{r}\frac{dw_{0}}{dr}+y^{*}\frac{1}{r}\phi \right)+\left(E_{1}\frac{d\phi }{dr}+\nu_{12}E_{2}\frac{1}{r}\phi \right)\int_{-\frac{h}{2}}^{\frac{h}{2}}\left.zf\left(z\right)dz\right)\} \end{array},$$

(28) $$\begin{array}{l} M_{\theta }^{L}=(1-l^{2}\nabla^{2})\{\frac{E_{2}}{1-\nu_{12}\nu_{21}}\left(\nu_{12}\right.\frac{h^{3}}{12}\left(-\frac{d^{2}w_{0}}{dr^{2}}+y^{*}\frac{d\phi }{dr}\right)+\\ \frac{h^{3}}{12}\left(-\frac{1}{r}\frac{dw_{0}}{dr}+y^{*}\frac{1}{r}\phi \right)+\left(\nu_{12}\frac{d\phi }{dr}+\frac{1}{r}\phi \right)\int_{-\frac{h}{2}}^{\frac{h}{2}}z\left.f\left(z\right)dz\right)\} \end{array},$$

(29) $$\begin{array}{l} R_{r}^{L}=(1-l^{2}\nabla^{2})\{\frac{1}{1-\nu_{12}\nu_{21}}\left(\left(E_{1}\left(\frac{du_{0}}{dr}+\frac{1}{2}{\left(\frac{dw_{0}}{dr}\right)}^{2}\right)+\nu_{12}E_{2}\frac{1}{r}u_{0}\right)\right.\int_{-\frac{h}{2}}^{\frac{h}{2}}f(z)dz\\ \;+\left(\nu_{12}E_{2}\left(-\frac{1}{r}\frac{dw_{0}}{dr}+y^{*}\frac{1}{r}\phi \right)+E_{1}\left(-\frac{d^{2}w_{0}}{dr^{2}}+y^{*}\frac{d\phi }{dr}\right)\right)\int_{-\frac{h}{2}}^{\frac{h}{2}}zf(z)dz+\\ \left(E_{1}\frac{d\phi }{dr}+\nu_{12}E_{2}\frac{1}{r}\phi \right)\left.\int_{-\frac{h}{2}}^{\frac{h}{2}}{\left(f(z)\right)}^{2}dz\right)\} \end{array},$$

(30) $$\begin{array}{l} R_{\theta }^{L}=(1-l^{2}\nabla^{2})\{\frac{E_{2}}{1-\nu_{12}\nu_{21}}\left(\left(\nu_{12}\left(\frac{du_{0}}{dr}+\frac{1}{2}{\left(\frac{dw_{0}}{dr}\right)}^{2}\right)+\frac{1}{r}u_{0}\right)\right.\int_{-\frac{h}{2}}^{\frac{h}{2}}f(z)dz+\\ \left(\nu_{12}\left(-\frac{d^{2}w_{0}}{dr^{2}}+y^{*}\frac{d\phi }{dr}\right)+\left(-\frac{1}{r}\frac{dw_{0}}{dr}+y^{*}\frac{1}{r}\phi \right)\right)\int_{-\frac{h}{2}}^{\frac{h}{2}}zf(z)dz+\\ \left(\nu_{12}\frac{d\phi }{dr}+\frac{1}{r}\phi \right)\left.\int_{-\frac{h}{2}}^{\frac{h}{2}}{\left(f(z)\right)}^{2}dz\right)\} \end{array},$$

(31) $$Q_{r}^{L}=(1-l^{2}\nabla^{2})\{G_{13}\phi y^{*}h+G_{13}\phi \int_{-\frac{h}{2}}^{\frac{h}{2}}\left(f^{\prime }(z)+y^{*}\right)dz\},$$

(32) $$R_{rz}^{L}=(1-l^{2}\nabla^{2})\{G_{13}\phi \int_{-\frac{h}{2}}^{\frac{h}{2}}{\left(f^{\prime }\left(z\right)\right)}^{2}dz+G_{13}y^{*}\phi \int_{-\frac{h}{2}}^{\frac{h}{2}}f^{\prime }\left(z\right)dz\}.$$

The equilibrium equations of a monolayer axisymmetric circular/annular nanoplate considering Pasternak and Winkler elastic foundations can be locally written as

(33) $$\delta u_{0}:N_{r}^{L}+r\frac{dN_{r}^{L}}{dr}-N_{\theta }^{L}=0,$$

(34) $$\delta \phi :y^{*}\left(r\frac{dM_{r}^{L}}{dr}+M_{r}^{L}-M_{\theta }^{L}-rQ_{r}^{L}\right)+R_{r}^{L}+r\frac{dR_{r}^{L}}{dr}-R_{\theta }^{L}-rR_{rz}^{L}=0,$$

(35) $$\begin{array}{l} \delta w_{0}:r\frac{d^{2}M_{r}^{L}}{dr^{2}}+2\frac{dM_{r}^{L}}{dr}-\frac{dM_{\theta }^{L}}{dr}+\left(1-\mu \nabla^{2}\right)\left(\left(-k_{w}w_{0}+k_{p}\nabla^{2}w_{0}\right)r\right.\\ \;+N_{\theta }^{L}\frac{dw_{0}}{dr}+\left.rN_{r}^{L}\frac{d^{2}w_{0}}{dr^{2}}\right)+\mu r\left(\left(\nabla^{2}N_{r}^{L}\right)\frac{d^{2}w_{0}}{dr^{2}}+\left.\left(\nabla^{2}N_{\theta }^{L}\right)\left(\frac{1}{r}\frac{dw_{0}}{dr}\right)\right)\right.=0 \end{array},$$

To derive the stability equations and determine the critical buckling force, the adjacent equilibrium method is used. Therefore, displacements are replaced by the following equations:

(36) $$\begin{array}{l} u=u_{0}+u_{1}\\ \phi =\phi_{0}+\phi_{1}\\ w=w_{0}+w_{1} \end{array}.$$

Therefore, for the resultants of force and moment

(37) $$\begin{array}{l} N_{r}^{L}=\left(N_{0r}^{L}+N_{1r}^{L}\right)\;\;\;,\;N_{\theta }^{L}=\left(N_{0\theta }^{L}+N_{1\theta }^{L}\right)\;\\ M_{r}^{L}=\left(M_{0r}^{L}+M_{1r}^{L}\right)\;\;\;,\;M_{\theta }^{L}=\left(M_{0\theta }^{L}+M_{1\theta }^{L}\right)\\ R_{r}^{L}=\left(R_{0r}^{L}+R_{1r}^{L}\right)\;\;\;\;,\;R_{\theta }^{L}=\left(R_{0\theta }^{L}+R_{1\theta }^{L}\right)\;\\ Q_{r}^{L}=\left(Q_{0r}^{L}+Q_{1r}^{L}\right)\;\;\;,\;R_{rz}^{L}=\left(R_{0rz}^{L}+R_{1rz}^{L}\right) \end{array}.$$

By substituting Equation (37) into equilibrium Equations (33)–(35), Equations (38)–(40) are obtained:

(38) $$\left(N_{0r}^{L}-N_{0\theta }^{L}+r\frac{dN_{0r}^{L}}{dr}\right)+\left(N_{1r}^{L}-N_{1\theta }^{L}+r\frac{dN_{1r}^{L}}{dr}\right)=0,$$

(39) $$\begin{array}{l} \left(y^{*}\left(r\frac{dM_{0r}^{L}}{dr}+M_{0r}^{L}-M_{0\theta }^{L}-rQ_{0r}^{L}\right)+R_{0r}^{L}-R_{0\theta }^{L}+r\frac{dR_{0r}^{L}}{dr}-rR_{0rz}^{L}\right)\\ +\left(y^{*}\left(r\frac{dM_{1r}^{L}}{dr}+M_{1r}^{L}-M_{1\theta }^{L}-rQ_{1r}^{L}\right)+R_{1r}^{L}-R_{1\theta }^{L}+r\frac{dR_{1r}^{L}}{dr}-rR_{1rz}^{L}\right)=0 \end{array},$$

(40) $$\begin{array}{l} \left(2\frac{dM_{0r}^{L}}{dr}-\frac{dM_{0\theta }^{L}}{dr}+r\frac{d^{2}M_{0r}^{L}}{dr^{2}}\right.+\left(1-\mu \nabla^{2}\right)\left(\left(-k_{w}w_{0}+k_{p}\nabla^{2}w_{0}\right)r\right.+\frac{dN_{1r}^{L}}{dr}r\frac{dw_{0}}{dr}\\ +N_{1r}^{L}\frac{dw_{0}}{dr}+rN_{1r}^{L}\frac{d^{2}w_{0}}{dr^{2}}+N_{0r}^{L}\frac{dw_{0}}{dr}+\frac{dN_{0r}^{L}}{dr}r\frac{dw_{0}}{dr}+rN_{0r}^{L}\frac{d^{2}w_{0}}{dr^{2}}\\ +\mu ((\nabla^{2}N_{1r}^{L})\frac{d^{2}w_{0}}{dr^{2}}+(\nabla^{2}N_{1\theta }^{L})(\frac{1}{r}\frac{dw_{0}}{dr}))+\mu ((\nabla^{2}N_{0r}^{L})\frac{d^{2}w_{0}}{dr^{2}}\left.+(\nabla^{2}N_{0\theta }^{L})\left.\left(\frac{1}{r}\frac{dw_{0}}{dr})\right)\right)\right)\\ +\left(2\frac{dM_{{}_{1r}}^{L}}{dr}-\frac{dM_{{}_{1\theta }}^{L}}{dr}+r\frac{d^{2}M_{1r}^{L}}{dr^{2}}\right.+\left(1-\mu \nabla^{2}\right)\left(\left(-k_{w}w_{1}+k_{p}\nabla^{2}w_{1}\right)r+\frac{dN_{{}_{1r}}^{L}}{dr}r\frac{dw_{1}}{dr}\right.\\ +N_{{}_{1r}}^{L}\frac{dw_{1}}{dr}+rN_{{}_{1r}}^{L}\frac{d^{2}w_{1}}{dr^{2}}+N_{{}_{0r}}^{L}\frac{dw_{1}}{dr}+\frac{dN_{{}_{0r}}^{L}}{dr}r\frac{dw_{1}}{dr}+rN_{{}_{0r}}^{L}\frac{d^{2}w_{1}}{dr^{2}}+\mu ((\nabla^{2}N_{1r}^{L})\frac{d^{2}w_{1}}{dr^{2}}\\ +(\nabla^{2}N_{1\theta }^{L})(\frac{1}{r}\frac{dw_{1}}{dr}))+\mu ((\nabla^{2}N_{0r}^{L})\frac{d^{2}w_{1}}{dr^{2}}+\left.\left(\nabla^{2}N_{0\theta }^{L})(\frac{1}{r}\frac{dw_{1}}{dr})\right)\right)=0 \end{array}.$$

In Equations (38)–(40), by omitting the terms that have only zero subscript and setting terms related to the preload state equal to zero and considering the uniform compressive load, we have

(41) $$N_{0r}^{L}=N_{0\theta }^{L}=-\bar{N}.$$

Therefore, the stability equations in terms of local stresses are written in the form of Equations (42)–(44):

(42) $$N_{{}_{1r}}^{L}-N_{{}_{1\theta }}^{L}+r\frac{\partial N_{{}_{1r}}^{L}}{\partial r}=0,$$

(43) $$y^{*}\left(M_{{}_{1r}}^{L}-M_{{}_{1\theta }}^{L}-rQ_{{}_{1r}}^{L}+r\frac{dM_{{}_{1r}}^{L}}{dr}\right)+R_{{}_{1r}}^{L}-R_{{}_{1\theta }}^{L}+r\frac{dR_{{}_{1r}}^{L}}{dr}-rR_{{}_{1rz}}^{L}=0,$$

(44) $$\begin{array}{l} 2\frac{dM_{{}_{1r}}^{L}}{dr}-\frac{dM_{{}_{1\theta }}^{L}}{dr}+r\frac{d^{2}M_{{}_{1r}}^{L}}{dr^{2}}+\left(1-\mu \nabla^{2}\right)\left(\left(-k_{w}w_{1}+k_{p}\nabla^{2}w_{1}\right)r+\left(\frac{dN_{{}_{1r}}^{L}}{dr}r+N_{{}_{1r}}^{L}\right)\frac{dw_{1}}{dr}\right.\\ +rN_{{}_{1r}}^{L}\frac{d^{2}w_{1}}{dr^{2}}-\bar{N}\left(\frac{dw_{1}}{dr}+r\frac{d^{2}w_{1}}{dr^{2}}\right)+\mu \left(\left(\nabla^{2}N_{1r}^{L}\right)\frac{d^{2}w_{1}}{dr^{2}}\right.+\left.\left.\left(\nabla^{2}N_{1\theta }^{L}\right)\left(\frac{1}{r}\frac{dw_{1}}{dr}\right)\right)\right)=0 \end{array}.$$

### 2.1. Derivation of the Governing Equations for the Bilayer Annular/Circular Nanoplate

**Figure 2:**
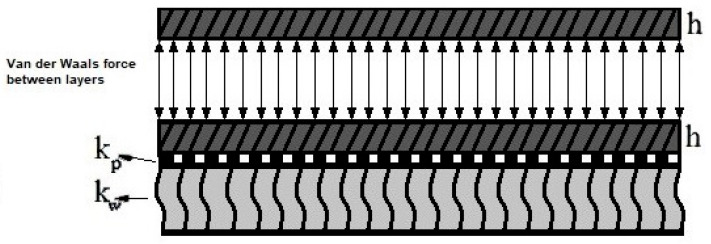
A schematic of the bilayer nanoplate considering the Pasternak and Winkler elastic foundations.

Numerous graphene layers can be employed to fix their weak buckling strengths. Therefore, graphene plates are set on top of each other by means of van der Waals bonds, generating graphene layers [42]. Van der Waals force is a general term used to explain the attraction of intermolecular forces among molecules (for more information please refer to Refs. [50,51]). Figure 2 shows a bilayer nanoplate (considering van der Waals forces between layers) on the Pasternak and Winkler elastic foundations.

Equilibrium equations considering the bilayer annular/circular nanoplate with the Pasternak–Winkler elastic foundation can be procured similar to the procedure used for the single-layer nanoplate. Furthermore, the annular/circular displacement fields of the axisymmetric bilayer nanoplate can be written as (*i* = 1 shows the top layer and *i* = 2 illustrates the bottom layer)

(45) $$U_{i}(r,\theta ,z)=u_{i}(r)-z\frac{dw_{i}(r)}{dr}+g\left(z\right)\phi_{i}(r)\;,\;i=1,2,$$

(46) $$V_{i}(r,\theta ,z)=0\;\;\;,\;i=1,2,$$

(47) $$W_{i}(r,\theta ,z)=w_{i}(r)\;,\;i=1,2.$$

In addition, the strain equations are the same as those procured in the single-layer annular/circular nanoplate. It should be noted that considering the minimum potential energy method to achieve the equilibrium equations (as well as boundary conditions) of the top and bottom layers, the following equations are considered:

(48) $$\delta \Omega_{1}=\int_{r_{i}}^{r_{o}}\int_{0}^{\tau }\left(k_{o}\left(w_{2}-w_{1}\right)\right)\delta w_{1}rdrd\theta ,$$

(49) $$\delta \Omega_{2}=\int_{r_{i}}^{r_{o}}\int_{0}^{\tau }\left(-k_{o}\left(w_{2}-w_{1}\right)-k_{w}w_{2}+k_{p}\nabla^{2}w_{2}\right)\delta w_{2}rdrd\theta ,$$

(50) $$\delta U=\underset{v}{∭}\sigma_{1}{}_{ij}^{NL}\delta \varepsilon_{1}{}_{ij}dv+\underset{v}{∭}\sigma_{2}{}_{ij}^{NL}\delta \varepsilon_{2}{}_{ij}dv\;\;i,j=r,\theta ,$$

(51) $$\delta \Pi =\delta U+\delta \Omega_{1}+\delta \Omega_{2}=0.$$

$k_{o}$ is the van der Waals stiffness constant between layers. Furthermore, the equilibrium equations in terms of local stresses for both layers can be mentioned as follows:

(52) $$\delta u_{i}:Ni_{r}^{L}+r\frac{dNi_{r}^{L}}{dr}-Ni_{\theta }^{L}=0\;,\;i=1,2,$$

(53) $$\delta \phi_{i}:y^{*}\left(r\frac{dMi_{r}^{L}}{dr}+Mi_{r}^{L}-Mi_{\theta }^{L}-rQi_{r}^{L}\right)+Ri_{r}^{L}+r\frac{dRi_{r}^{L}}{dr}-Ri_{\theta }^{L}-rRi_{rz}^{L}=0\;,\;i=1,2,$$

(54) $$\begin{array}{l} \delta w_{1}:r\frac{d^{2}M_{1}{}_{r}^{L}}{dr^{2}}+2\frac{dM_{1}{}_{r}^{L}}{dr}-\frac{dM_{1}{}_{\theta }^{L}}{dr}+\\ \left(1-\mu \nabla^{2}\right)\left(\left(k_{0}\left(w_{2}-w_{1}\right)\right)r+N_{1}{}_{\theta }^{L}\frac{dw_{1}}{dr}\right.+rN_{1}{}_{r}^{L}\frac{d^{2}w_{1}}{dr^{2}}-\bar{N}\left(\frac{dw_{1}}{dr}+r\frac{d^{2}w_{1}}{dr^{2}}\right)\\ +\mu \left(\left(\nabla^{2}N_{1r}^{L}\right)\frac{\partial^{2}w_{1}}{\partial r^{2}}\right.+\left.\left.\left(\nabla^{2}N_{1\theta }^{L}\right)\left(\frac{1}{r}\frac{\partial w_{1}}{\partial r}\right)\right)\right)=0 \end{array},$$

(55) $$\begin{array}{l} \delta w_{2}:r\frac{d^{2}M_{2}{}_{r}^{L}}{dr^{2}}+2\frac{dM_{2}{}_{r}^{L}}{dr}-\frac{dM_{2\theta }^{L}}{dr}\\ +\left(1-\mu \nabla^{2}\right)\left(\left(-k_{0}\left(w_{2}-w_{1}\right)-k_{w}w_{2}+k_{p}\nabla^{2}w_{2}\right)r+N_{2\theta }^{L}\frac{dw_{2}}{dr}+rN_{2r}^{L}\frac{d^{2}w_{2}}{dr^{2}}\right.\\ -\bar{N}\left(\frac{dw_{2}}{dr}+r\frac{d^{2}w_{2}}{dr^{2}}\right)+\mu \left(\left(\nabla^{2}N_{2r}^{L}\right)\frac{\partial^{2}w_{2}}{\partial r^{2}}\right.+\left.\left.\left(\nabla^{2}N_{2\theta }^{L}\right)\left(\frac{1}{r}\frac{\partial w_{2}}{\partial r}\right)\right)\right)=0 \end{array}.$$

In addition, the stability equations can be presented for the thermal buckling of circular and annular plates. Moreover, the only difference between the equations of thermal buckling compared to mechanical buckling is in the strains. Consequently, the stress and moment resultants change. The nonlinear strains, taking into account von Karman’s assumptions, can be written as Equations (56)–(58):

(56) $$\varepsilon_{r}=\frac{du}{dr}-z\frac{d^{2}w}{dr^{2}}+g\left(z\right)\frac{d\phi }{dr}+\frac{1}{2}{\left(\frac{dw}{dr}\right)}^{2}-\alpha_{11}\Delta T,$$

(57) $$\varepsilon_{\theta }=\frac{u}{r}-\frac{z}{r}\frac{dw}{dr}+\frac{1}{r}g\left(z\right)\phi -\alpha_{22}\Delta T,$$

(58) $$\gamma_{rz}=\phi \frac{dg\left(z\right)}{dz}.$$

The local stress resultants (considering thermal effects) according to displacements are as follows:

(59) $$\begin{array}{l} N_{r}^{L}=\frac{1}{1-\nu_{12}\nu_{21}}\left(E_{1}h\right.\left(\frac{du}{dr}+\frac{1}{2}{\left(\frac{dw}{dr}\right)}^{2}\right)+\nu_{12}E_{2}h\frac{1}{r}u+\left(E_{1}\frac{d\phi }{dr}+\nu_{12}E_{2}\frac{1}{r}\phi \right)\int_{-\frac{h}{2}}^{+\frac{h}{2}}f\left(z\right)\left.dz\right)\\ -\frac{h\Delta T\left(E_{2}\alpha_{22}\nu_{12}+E_{1}\alpha_{11}\right)}{1-\nu_{12}\nu_{21}} \end{array},$$

(60) $$\begin{array}{l} N_{\theta }^{L}=\frac{1}{1-\nu_{12}\nu_{21}}\left(\nu_{12}E_{2}h\right.+\left(\frac{du}{dr}+\frac{1}{2}{\left(\frac{dw}{dr}\right)}^{2}\right)+E_{2}h\frac{1}{r}u+\left(\nu_{12}E_{2}\frac{d\phi }{dr}+E_{2}\frac{1}{r}\phi \right)\int_{-\frac{h}{2}}^{+\frac{h}{2}}f\left(z\right)\left.dz\right)\\ -\frac{E_{2}h\Delta T\left(\alpha_{11}\nu_{12}+E_{1}\alpha_{22}\right)}{1-\nu_{12}\nu_{21}} \end{array}.$$

In order to derive the stability equations from the equilibrium equations and determine the critical buckling temperature using the adjacent equilibrium technique, the equilibrium equations are obtained in the form of Equations (38)–(40). In those equations, by ignoring the terms that only have a zero subscript, zeroing the terms related to the preload state, and knowing that in thermal buckling

(61) $$N_{{}_{0r}}^{L}=N_{{}_{0\theta }}^{L}=-{\bar{N}}_{T}=\frac{h\Delta T\left(E_{2}\alpha_{22}\nu_{12}+E_{1}\alpha_{11}\right)}{1-\nu_{12}\nu_{21}}.$$

Stability equations in terms of local stresses are written in the form of Equations (62)–(64):

(62) $$N_{{}_{1r}}^{L}-N_{{}_{1\theta }}^{L}+r\frac{\partial N_{{}_{1r}}^{L}}{\partial r}=0,$$

(63) $$y^{*}\left(M_{{}_{1r}}^{L}-M_{{}_{1\theta }}^{L}-rQ_{{}_{1r}}^{L}+r\frac{\partial M_{{}_{1r}}^{L}}{\partial r}\right)+R_{{}_{1r}}^{L}-R_{{}_{1\theta }}^{L}+r\frac{\partial R_{{}_{1r}}^{L}}{\partial r}-rR_{{}_{1rz}}^{L}=0,$$

(64) $$\begin{array}{l} 2\frac{\partial M_{{}_{1r}}^{L}}{\partial r}-\frac{\partial M_{{}_{1\theta }}^{L}}{\partial r}+r\frac{\partial^{2}M_{{}_{1r}}^{L}}{\partial r^{2}}+\left(1-\mu \nabla^{2}\right)\left(\left(\frac{\partial N_{{}_{1r}}^{L}}{\partial r}r+N_{{}_{1r}}^{L}\right)\right.\frac{\partial w_{1}}{\partial r}+rN_{{}_{1r}}^{L}\frac{\partial^{2}w_{1}}{\partial r^{2}}\\ -{\bar{N}}_{T}\left(\frac{\partial w_{1}}{\partial r}+r\frac{\partial^{2}w_{1}}{\partial r^{2}}\right)+\mu \left(\left(\nabla^{2}N_{1r}^{L}\right)\frac{\partial^{2}w_{1}}{\partial r^{2}}\right.+\left.\left.\left(\nabla^{2}N_{1\theta }^{L}\right)\left(\frac{1}{r}\frac{\partial w_{1}}{\partial r}\right)\right)\right)=0 \end{array}.$$

### 2.2. Equations of Thermal Stability for a Bilayer Annular/Circular Plate

The stability equations in terms of local stresses (considering the first layer (*i* = 1) and the second layer (*i* = 2)) can be written as follows:

(65) $$\delta u_{i}:Ni_{r}^{L}+r\frac{dNi_{r}^{L}}{dr}-Ni_{\theta }^{L}=0\;,\;i=1,2,$$

(66) $$\delta \phi_{i}:y^{*}\left(r\frac{dMi_{r}^{L}}{dr}+Mi_{r}^{L}-Mi_{\theta }^{L}-rQi_{r}^{L}\right)+Ri_{r}^{L}+r\frac{dRi_{r}^{L}}{dr}-Ri_{\theta }^{L}-rRi_{rz}^{L}=0\;,\;i=1,2,$$

(67) $$\begin{array}{l} \delta w_{1}:r\frac{d^{2}M_{1r}^{L}}{dr^{2}}+2\frac{dM_{1r}^{L}}{dr}-\frac{dM_{1\theta }^{L}}{dr}+\left(1-\mu \nabla^{2}\right)\;\left(\left(k_{0}\left(w_{2}-w_{1}\right)\right)r+N_{1\theta }^{L}\frac{dw_{1}}{dr}\right.\\ +rN_{1r}^{L}\frac{d^{2}w_{1}}{dr^{2}}-{\bar{N}}_{T}\left(\frac{dw_{1}}{dr}+r\frac{d^{2}w_{1}}{dr^{2}}\right)+\mu \left(\left(\nabla^{2}N_{1r}^{L}\right)\frac{\partial^{2}w_{1}}{\partial r^{2}}\right.+\left.\left.\left(\nabla^{2}N_{1\theta }^{L}\right)\left(\frac{1}{r}\frac{\partial w_{1}}{\partial r}\right)\right)\right)=0 \end{array},$$

(68) $$\begin{array}{l} \delta w_{2}:r\frac{d^{2}M_{2r}^{L}}{dr^{2}}+2\frac{dM_{2r}^{L}}{dr}-\frac{dM_{2\theta }^{L}}{dr}+\left(1-\mu \nabla^{2}\right)\left(\left(-k_{0}\left(w_{2}-w_{1}\right)-k_{w}w_{2}+k_{p}\nabla^{2}w_{2}\right)r\right.\\ +N_{2\theta }^{L}\frac{dw_{2}}{dr}+rN_{2r}^{L}\frac{d^{2}w_{2}}{dr^{2}}-{\bar{N}}_{T}\left(\frac{dw_{2}}{dr}+r\frac{d^{2}w_{2}}{dr^{2}}\right)+\mu \left(\left(\nabla^{2}N_{2r}^{L}\right)\frac{\partial^{2}w_{2}}{\partial r^{2}}\right.+\left.\left.\left(\nabla^{2}N_{2\theta }^{L}\right)\left(\frac{1}{r}\frac{\partial w_{2}}{\partial r}\right)\right)\right)=0 \end{array}.$$

### 2.3. Boundary Conditions

The boundary conditions for the annular/circular plate can be assumed as

Clamped (C):

(69) $$u=w=\phi =\frac{dw}{dr}=0.$$

Simply supported (S):

(70) $$u=w=M_{r}=R_{r}=0.$$

Free (F):

(71) $$N_{r}=M_{r}=R_{r}=Q_{r}=0\;.$$

### 2.4. Nondimensional Assumptions

Because of the very small values that exist on the nanoscale, and for the ease of calculations, Equation (72) is introduced to convert the equations of the nanoplate nondimensional:

(72) $$\begin{array}{l} u^{*}=\frac{u_{0}}{h};w^{*}=\frac{w_{0}}{r_{o}};\phi^{*}=\phi \;;\;\psi^{*}=\psi ;N_{r}^{*}=\frac{N_{r}}{E_{1}h};\;N_{\theta }^{*}=\frac{N_{\theta }}{E_{1}h};\\ \;Q_{r}^{*}=\frac{Q_{r}}{E_{1}h}\;;Q_{\theta }^{*}=\frac{Q_{\theta }}{E_{1}h};M_{r}^{*}=\frac{M_{r}}{E_{1}h^{2}}\;;\;M_{\theta }^{*}=\frac{M_{\theta }}{E_{1}h^{2}}\;;\;\nabla^{*}{}^{2}=\frac{d^{2}}{dr^{*}{}^{2}}+\frac{1}{r^{*}}\frac{d}{dr^{*}};\\ R_{r}^{*}=\frac{R_{r}}{E_{1}h^{2}};R_{\theta }^{*}=\frac{R_{\theta }}{E_{1}h^{2}};R_{rz}^{*}=\frac{R_{rz}}{E_{1}h};\;r^{*}=\frac{r}{r_{o}}\;;\;z^{*}=\frac{z}{h}\;;\;\delta =\frac{h}{r_{o}};k_{w}^{*}=\frac{k_{w}r_{o}}{E_{1}}\;;k_{p}^{*}=\frac{k_{p}}{E_{1}r_{o}} \end{array}$$

## 3. Numerical Procedure

DQM is one of the most efficient and elegant techniques for the numerical solution of partial differential equations used by many authors (which are briefly explained in the Introduction). This method is obtained from the quadratic integration technique, which expresses the integral at one point in the direction of the domain and relies on all points along that direction. Moreover, the value of dependency can be obtained using weight constants:

(73) $$\int_{a}^{b}f(r)dr=\sum_{k=1}^{n}w_{k}f_{k}$$

where, $w_{1},w_{2},\ldots ,w_{n}$ as well as $f_{1},f_{2},\ldots ,f_{n}$ can be defined as weight constants and function values at discrete points, respectively. Belman et al. [52] (regarding quadratic integration) proposed that the derivative at one node of the function domain can be based on the function values at the entire nodes of the domain via weight constants.

(74) $${\left.\frac{df}{dr}\right|}_{r_{i}}=\sum_{j=1}^{N}A_{ij}f\left(r_{j}\right)\;,\;i=1,2,\ldots ,N$$

in which $A_{ij}$ and *N* are the weight constant and the total number of nodes in the direction of *r*, respectively. For the first-order derivative, the weighting constants can be gained as

(75) $$A_{ij}^{\left(1\right)}=\frac{P\left(r_{i}\right)}{\left(r_{i}-r_{j}\right)P\left(r_{j}\right)},$$

(76) $$P\left(r_{i}\right)=\overset{N}{\underset{j=1}{\prod }}\left(r_{i}-r_{j}\right)\;,\;i\neq j,$$

(77) $$A_{ii}^{\left(1\right)}=-\sum_{k=1}^{N}A_{ik}^{\left(1\right)}\;\;\;,\;i\neq k.$$

For higher-order derivatives, the following equation can be defined as

(78) $${\left.\frac{d^{\left(n\right)}f}{dr^{\left(n\right)}}\right|}_{r_{i}}=\sum_{j=1}^{N}A_{ij}^{\left(n\right)}f(r_{j})\;,\;i=1,\ldots ,N.$$

For the second- and higher-order derivatives, the weighting constants can be defined as follows:

(79) $$A_{ij}^{\left(n\right)}=n\left[A_{ij}^{\left(1\right)}A_{ii}^{\left(n-1\right)}-\frac{A_{ij}^{\left(n-1\right)}}{\left(r_{i}-r_{j}\right)}\right]\;,\;i\neq j,$$

(80) $$A_{ii}^{\left(n\right)}=-\sum_{j=1,\neq i}^{N}A_{ij}^{\left(n\right)}\;,\;i,j=1\ldots N.$$

In this study, the grid points are distributed according to the Chebyshev–Gauss–Lubato distribution as follows:

(81) $$r_{i}=\frac{r_{i}+r_{o}}{2}-\cos\left(\left(\frac{i-1}{N-1}\right)\pi \right)\left(\frac{r_{o}-r_{i}}{2}\right),\;i=1\ldots N$$

where $r_{i}$ and $r_{o}$ are nodes at the start and end of the function.

It should be noted that for each node (considering a single-layer plate) there are three equations and four unknowns, whose unknowns are displacement components of the plate $\left(w_{0},\varphi ,u_{0}\right)$ and critical buckling load in the case of mechanical/thermal axial symmetric analysis. In other words, there are 27 equations and 28 unknowns for 9 nodes. The desired unknown (mechanical buckling critical load or thermal buckling critical term) is obtained after applying the DQM. Therefore, by inserting Equations (25)–(32) (which are the resultants in terms of displacements) in the stability equations, the stability equations in terms of displacements are obtained. Then, by using Equation (74) (for first-order derivatives) or Equation (78) (for higher-order derivatives) and by using appropriate weighting constants (Equations (75)–(77) for the first-order derivative or Equations (79)–(80) for higher derivatives) the discretized form of equations will be obtained in terms of displacements. The matrix form of these equations can be written as follows:

(82) $${\left[Cef\right]}_{3N\times 3N}\left\{\left.\begin{array}{l} u_{0}^{*1}\\ \varphi^{*1}\\ w_{0}^{*1}\\ .\\ .\\ u_{0}^{*N}\\ \varphi^{*N}\\ w_{0}^{*N} \end{array}\right\}\right.=0.$$

$\left[Cef\right]$ is the matrix form of coefficients of equations. For *N* nodes, the matrix form of equations includes 3*N algebraic equations and 3*N + 1 unknowns. Since solving these algebraic equations and unknowns are long and time-consuming, Maple software 2023 was used to solve it. So, in the computer program, the determinant of the matrix of coefficients should be equal to zero $\left(\left|Cef\right|=0\right)$ to obtain the dimensionless critical load of mechanical buckling or the dimensionless term of thermal buckling. Then, an equation will be obtained that contains a Polynomial in terms of buckling load. By solving it, and in order to find the critical buckling load, the minimum value should be chosen.

Also, for a better understanding of applying DQM, an example of the discrete form of the equations can be seen in Appendix A.

## 4. Results and Discussion

In this part, various factors are studied to identify their effect on the nondimensional buckling load of the annular/circular nanoplate. For this purpose, HSDT, FSDT, and the nonlocal strain gradient theory are utilized with the aid of DQM. Moreover, to validate the procedure, the current results are compared with the results of references. Also, the following assumptions are considered (if not mentioned in the text):

(83) $$\begin{array}{l} k_{w}=1.13\left(\mathrm{GPa}/\mathrm{nm}\right)\;;\;k_{p}=1.13\left(\mathrm{Pa}.m\right)\;,k_{0}=45\left(\mathrm{GPa}/\mathrm{nm}\right),\;r_{o}=5\left(\mathrm{nm}\right),\\ E_{1}=1765\;(\mathrm{GPa}),\;E_{2}=1588\;(\mathrm{GPa}),\nu_{12}=0.3,\;\nu_{21}=0.27,\;h=0.34\;(\mathrm{nm}).\; \end{array}$$

**Figure 3:**
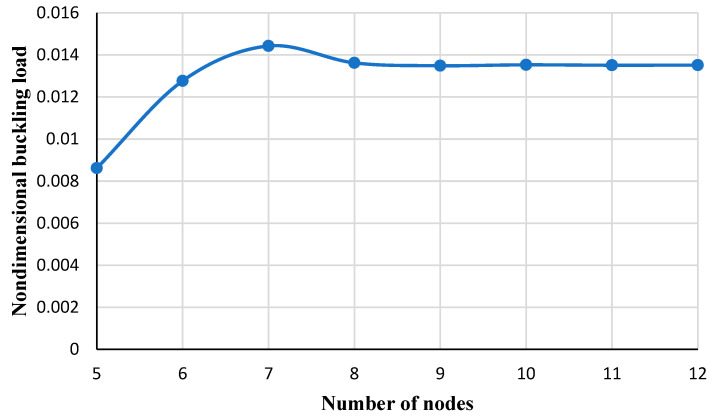
The effect of the number of nodes on the dimensionless mechanical buckling load.

Figure 3 reveals the influence of the number of nodes employed in the DQM to achieve the results of this paper. As can be seen, after nine nodes the appropriate convergence is gained. So, the number of nine nodes is considered to obtain the numerical results.

To verify accuracy and validity, the present results (gained using both FSDT and HSDT) in the clamped boundary condition are compared with the reference, which shows good accuracy (Table 2). In addition, it can be concluded that by increasing the radius of the plate, the nondimensional buckling loads are decreased.

Table 3 compares the nondimensional buckling load gained by HSDT by considering various shape functions for a circular nanoplate at clamped as well as simply supported boundary conditions. Therefore, different functions are used to distribute the shear stress along the thickness, which can be summarized as:

(84) $$g_{1}\left(z\right)=\frac{h}{\pi }\sin\left(\frac{\pi z}{h}\right),$$

(85) $$g_{2}\left(z\right)=-\frac{4}{3h^{2}}z^{3}+z,$$

(86) $$g_{3}\left(z\right)=h\sinh\left(\frac{z}{h}\right)-z\cosh\left(\frac{1}{2}\right),$$

(87) $$g_{4}\left(z\right)=ze^{-2{\left(\frac{z}{h}\right)}^{2}},$$

(88) $$g_{5}\left(z\right)=-\frac{5}{3h^{2}}z^{3}+\frac{5}{4}z.$$

From Table 3, it can be deduced that using these shape functions results in obtaining almost the same values of dimensionless buckling loads for both clamped and simply supported boundary conditions.

**Figure 4:**
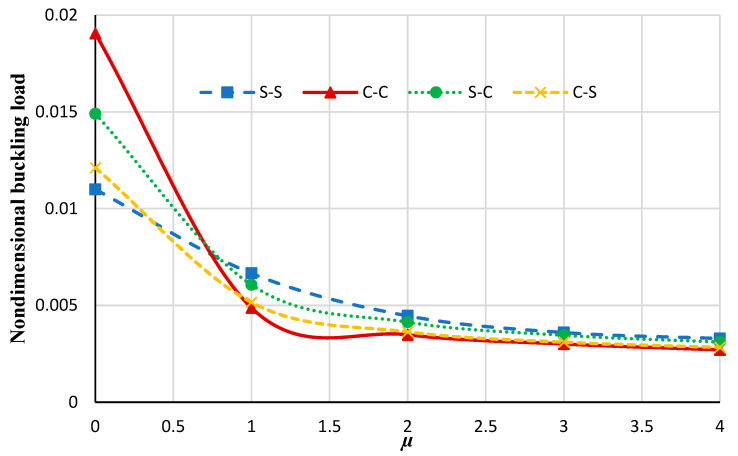
The nondimensional buckling load of the annular nanoplate with respect to the nonlocal parameter in diverse boundary conditions.

Figure 4 illustrates the changes in the nondimensional buckling loads versus the nonlocal constant for the annular nanoplate at various boundary conditions. It can be seen that by increasing the nonlocal parameter the non-dimensional buckling load decreases. This may be persuaded by increasing the nonlocal parameter; the stiffness of the nanoplate is increased and, therefore, the nondimensional buckling of the plate is reduced. Also, by increasing the rigidity of the boundary condition, increasing the nonlocal parameter results in a significant decrease in the nondimensional buckling load. Moreover, as the nonlocal coefficient increases the effect of the boundary condition on the nondimensional load decreases.

**Figure 5:**
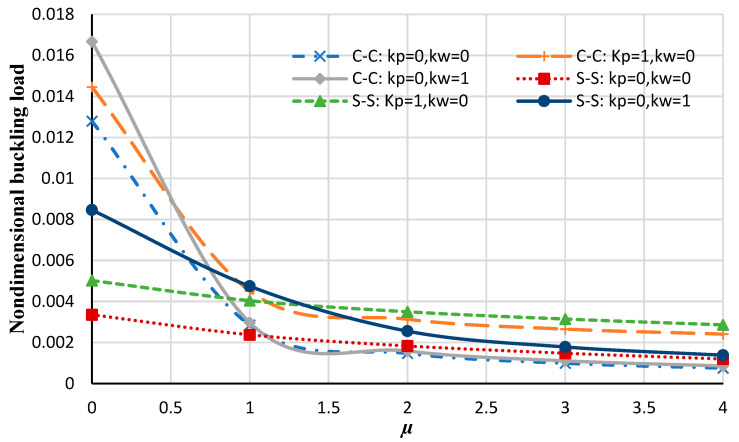
Nondimensional buckling loads of the annular nanoplate with respect to the nonlocal parameter for different values of elastic foundations at various boundary conditions (including C-C and S–S).

**Figure 6:**
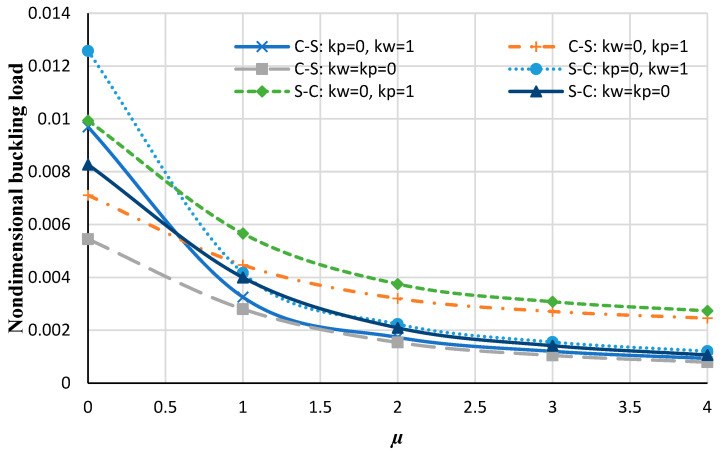
The nondimensional buckling load of the annular nanoplate with respect to the nonlocal parameter for different values of elastic foundations at various boundary conditions (including C–S and S–C).

From Figure 5 and Figure 6, it is distinguished that generally increasing the elastic foundation results in an increase in the nondimensional buckling load, which is due to the fact that assuming an elastic medium results in a stiffer structure. Also, it is noticed that in local analysis, the nondimensional buckling loads with the Winkler foundation are higher than values with the Pasternak foundation, but by increasing the nonlocal parameter the Pasternak foundation has more values of nondimensional buckling loads. Moreover, In the local analysis, by increasing the flexibility of the boundary condition, the nondimensional buckling load decreases, but it can be noticed in Figure 5 and Figure 6, by increasing the rigidity of the boundary condition and increasing the nonlocal coefficient, the nondimensional buckling load decreases. This can highlight that in nonlocal analyses, the influence of the nonlocal coefficient on the buckling analysis of nanoplate is more significant than the type of boundary conditions or elastic foundations.

**Figure 7:**
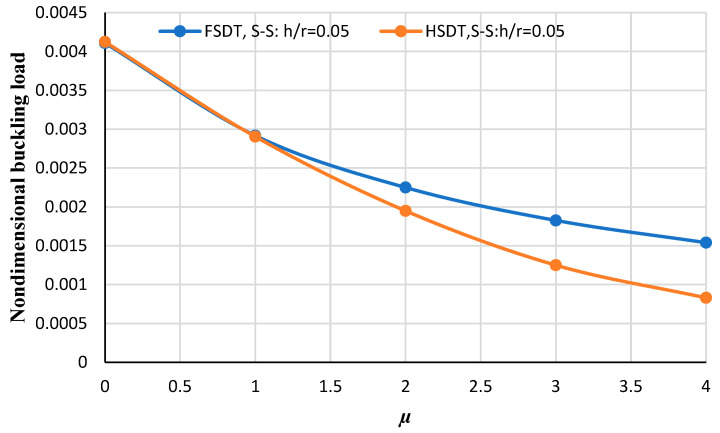
The nondimensional buckling load of the annular nanoplate with respect to the nonlocal parameter for FSDT and HSDT considering *h/r* = 0.05 for the simply supported boundary condition.

**Figure 8:**
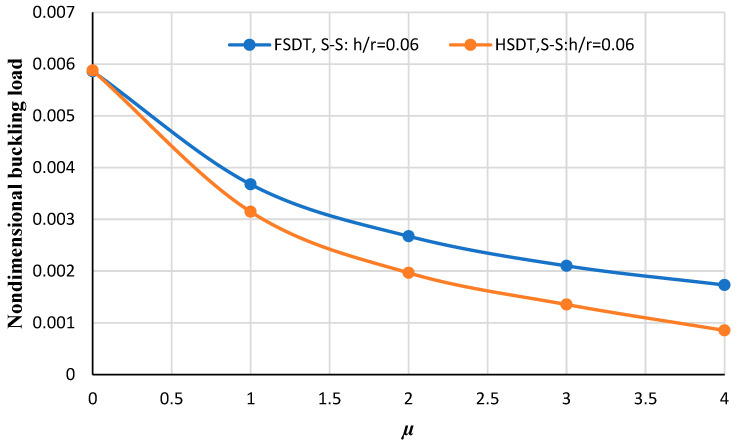
The nondimensional buckling load of the annular nanoplate with respect to the nonlocal parameter for FSDT and HSDT considering *h/r* = 0.06 for the simply supported boundary condition.

Figure 7 and Figure 8 illustrate the nondimensional buckling load versus the nonlocal parameter obtained from HSDT and FSDT for two thickness-to-radius ratios including *h/r* = 0.05 and *h/r* = 0.06. As can be deduced by increasing the nonlocal parameter, the non-dimensional buckling load obtained from HSDT and FSDT differ significantly. Also, by comparing these two figures, it can be seen that in the higher thickness-to-radius ratios the difference between FSDT and HSDT increases more significantly.

**Figure 9:**
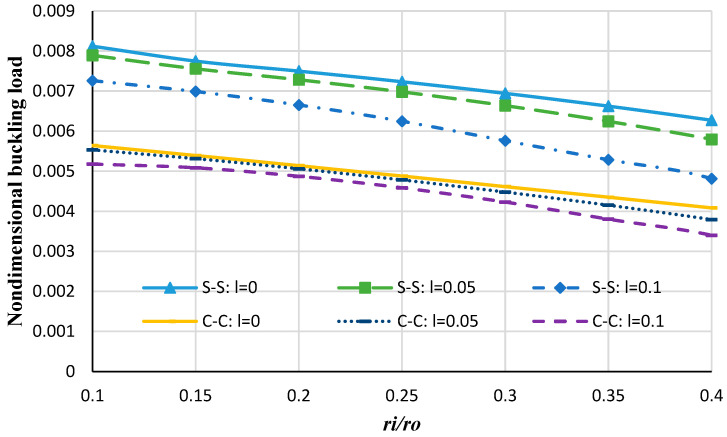
The nondimensional buckling load of the annular nanoplate versus the radius ratios.

Figure 9 shows the nondimensional buckling load versus the radius ratios (inner radius-to-outer radius ratios) for different boundary conditions and considering different values of strain gradients. It can be concluded that by increasing radius ratios the buckling loads decrease. Also, increasing the strain gradient results in a decrease in buckling loads which specifies that inclusion of the strain gradient coefficient makes the plate stiffer than that of the classical plate [54].

### 4.1. Thermal Analysis

**Figure 10:**
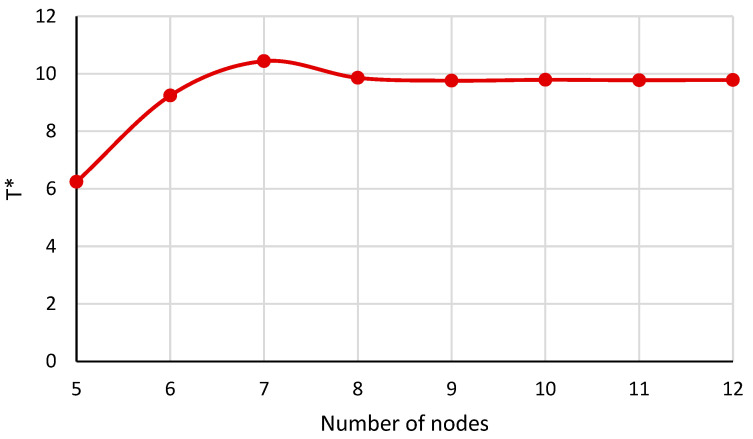
The critical buckling temperature for the annular nanoplate with respect to the number of nodes.

In this section, to validate the research, the current results of thermal buckling of circular/annular plates are compared with the results of valid reference, and then the results of the relevant diagrams are presented. Figure 10 reveals the effect of the number of nodes employed in the DQM to achieve the results of thermal analysis. It is noted that $T^{*}$ is the critical buckling temperature, which is considered as follows:

(89) $$T^{*}=10^{3}\alpha^{T}\Delta T.$$

ΔT is the difference between the critical buckling temperature and the reference temperature. The coefficient of thermal expansion is considered as

(90) $$\alpha^{T}=1.1\times 10^{-6}\left(K^{-1}\right)\;.$$

In order to validate the numerical results of the present article and the solution technique and compare them with the results of Ref. [55], the dimensionless thermal buckling parameter ($\lambda_{T}$) is defined as follows:

(91) $$\lambda_{T}=12\left(1+\nu \right)\alpha^{T}\;\Delta T{\left(\frac{r_{o}}{h}\right)}^{2}.$$

In this comparison, the dimensionless parameter of the thermal buckling of the isotropic graphene plate for the annular nanoplate assuming various boundary conditions, radius ratios, and thickness-to-radius ratios is given in Table 4. According to this table, it is clear that in smaller thickness-to-radius ratios, the results agree well with the results of Ref. [43], and as the $h/r_{o}$ and $r_{i}/r_{o}$ ratios increase, a small amount of difference is observed. Moreover, the difference between the results is caused by the different types of theories.

To better observe the differences between the present study with the reference [55], the percentage errors are gathered in Table 5, which can be defined as follows:

(92) $$E_{1}(\%)=\left|\frac{E_{\mathrm{HSDT}}-E_{\mathrm{Ref}}}{E_{\mathrm{Ref}}}\right|\times 100,\;E_{2}(\%)=\left|\frac{E_{\mathrm{FSDT}}-E_{\mathrm{Ref}}}{E_{\mathrm{Ref}}}\right|\times 100$$

where $E_{1}(\%)$ and $E_{2}(\%)$ represent the HSDT and FSDT errors compared to the reference’s values, respectively. Also, $E_{\mathrm{HSDT}}$, $E_{\mathrm{FSDT}}$, and $E_{\mathrm{Ref}}$ represent values of the dimensionless thermal buckling parameter $\left(\lambda_{T}\right)$ gained by the present study HSDT, FSDT, and the reference (obtained from Table 4), respectively.

**Figure 11:**
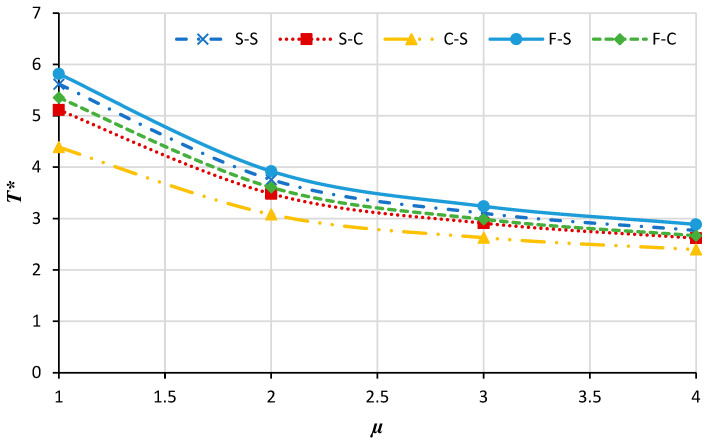
The critical buckling temperature for the annular nanoplate versus the nonlocal parameter.

Figure 11 shows the critical buckling temperature of the annular nanoplate versus the nonlocal parameter for different boundary conditions. As can be distinguished, the critical buckling temperature decreases with the rise of the nonlocal parameter (which has the same trend as the mechanical analysis).

Finally, by comparing the equations and checking the results for both thermal and mechanical analyses, it is found that with the following equation, thermal and mechanical buckling analyses can be related to each other:

(93) $$T^{*}=N^{*}\lambda \;,\;\lambda =\frac{\left(1-\nu_{12}\nu_{21}\right)}{\alpha^{T}(1+\frac{E_{2}}{E_{1}}\nu_{12})}.$$

It is noted that in Equation (93), $T^{*}$ is critical buckling temperature and $N^{*}$ is mechanical load. In other words, the results of the thermal buckling can be gained using Equation (93) which can save the time of computations.

### 4.2. Bilayer Analysis

**Figure 12:**
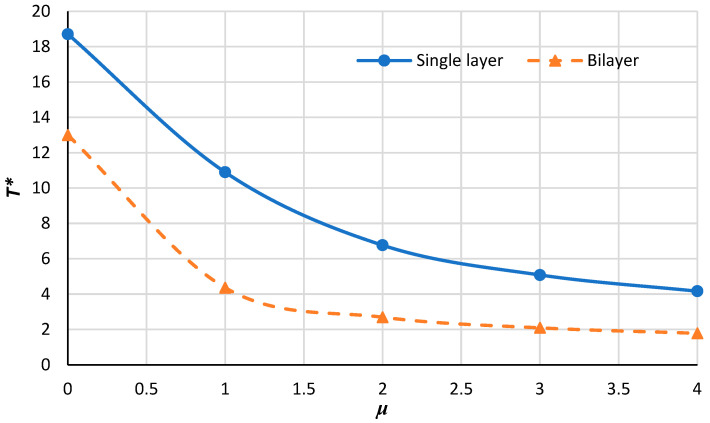
Critical buckling temperature for single-layer and bilayer annular nanoplate with respect to the nonlocal parameter for the C–C condition.

**Figure 13:**
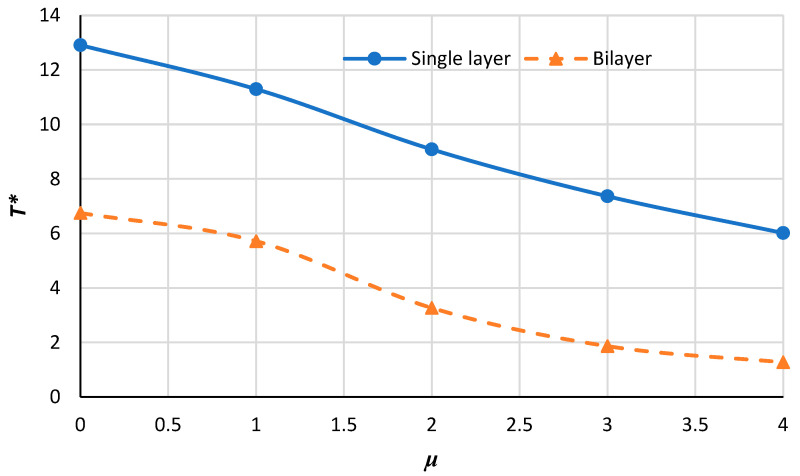
Critical buckling temperature for single-layer and bilayer annular nanoplate versus the nonlocal parameter for S–S condition.

**Figure 14:**
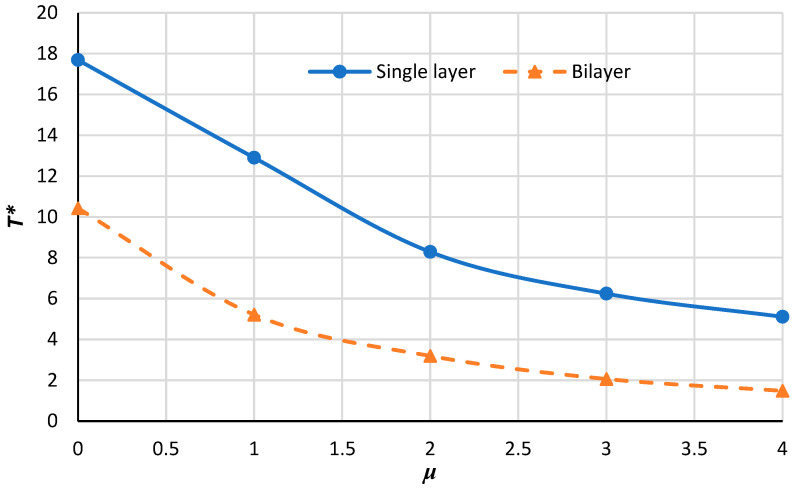
Critical buckling temperature for single-layer and bilayer annular nanoplate versus the nonlocal parameter for S–C condition.

**Figure 15:**
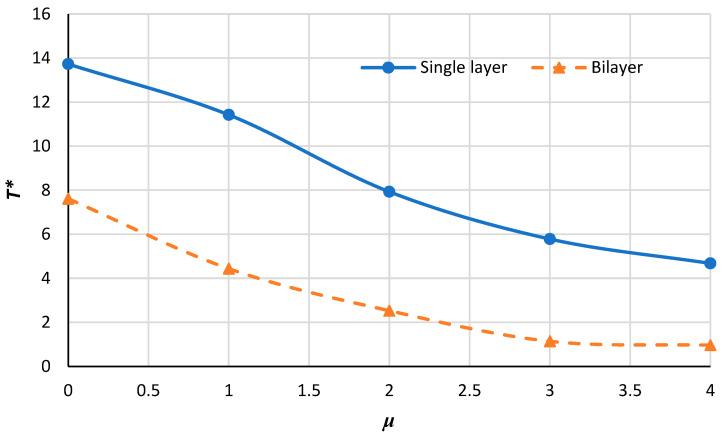
Critical buckling temperature for single-layer and bilayer annular nanoplate versus the nonlocal parameter for C–S condition.

Figure 12, Figure 13, Figure 14 and Figure 15 demonstrate the variation in the critical buckling temperature in terms of nonlocal constants for the annular bilayer nanoplate with a thickness of *h* (0.34 nm) and the single-layer nanoplate considering a thickness of 2*h (0.68 nm) for different boundary conditions. In fact, this part studies the probability of replacing a single-layer nanoplate (considering double thickness) with a bilayer nanoplate. It can be concluded that a bilayer nanoplate cannot be considered a single-layer nanoplate with the same thickness (as the bilayer nanoplate) for obtaining the exact results of the buckling analysis. Furthermore, the results obtained from the equivalent single-layer nanoplate with double thickness overestimate the real results gained using a bilayer nanoplate. This can be justified because of the van der Waals interaction that exists between the layers of bilayer nanoplate, which is an approximately weak force. If the buckling loads are strong enough, then molecules break free of the van der Waals forces that hold them together. Thus, the results of a single-layer plate are not similar to those of a bilayer plate.

## 5. Conclusions

In this paper, the nonlinear mechanical and thermal buckling of the circular and annular (single layer/bilayer) nanoplate using the nonlocal strain gradient theory considering both HSDT and FSDT is carried out. DQM is employed to solve the governing equations of the annular/circular nanoplate. Also, the results were compared with other references that showed good agreement. Some of the results of this article can be summarized as follows:

- In the higher thickness-to-radius ratios, the difference between FSDT and HSDT increases more significantly.
- The influence of the nonlocal coefficient on the buckling analysis of circular/annular nanoplate is more significant than the types of boundary conditions or elastic foundations.
- A bilayer nanoplate cannot be considered an equivalent single-layer nanoplate (with the same thickness as the bilayer nanoplate) to obtain the results of the buckling analysis of nanoplate. Furthermore, the results obtained from the equivalent single-layer nanoplate with double thickness overestimate the real results gained using a bilayer nanoplate.

## Figures and Tables

**Table 1:** Recommended functions used in HSDT by various authors.

Model	g(z) Function
Ambartsumian [42]	$-\frac{1}{6}z^{3}+\frac{h^{2}}{8}z$
Reddy [43]	$-\frac{4}{3h^{2}}z^{3}+z$
Reissner [44]	$-\frac{5}{3h^{2}}z^{3}+\frac{5}{4}z$
Touratier [45]	$\frac{h}{\pi }\sin\left(\frac{\pi z}{h}\right)$
Soldatos [46]	$h\sinh\left(\frac{z}{h}\right)-z\cosh\left(\frac{1}{2}\right)$
Aydogdu [47]	$ze^{-2{\left(\frac{z}{h}\right)}^{2}}$
Mantari [48]	$\frac{h}{\pi }\left(\sin\left(\frac{\pi z}{h}\right)e^{m\cos\left(\frac{\pi z}{h}\right)}+m\frac{\pi }{h}z\;\right)\;\;\;,\;\;\;\;m\geq 0$

**Table 2:** Comparison of the nondimensional buckling loads of the circular nanoplate gained by the present study with a reference.

Radius	Reference	The Percentage of the Nondimensional Buckling Loads				
		*μ* = 0	*μ* = 0.25	*μ* = 1	*μ* = 2.25	*μ* = 4
**4**	Ref. [53]	0.9430	0.7671	0.4918	0.3077	0.2019
	Present FSDT results	0.9353	0.7554	0.4794	0.3008	0.1943
	Present HSDT results	0.9211	0.7435	0.3433	0.2432	0.0913
**6**	Ref. [53]	0.4191	0.3803	0.2977	0.2186	0.1593
	Present FSDT results	0.4222	0.3822	0.2975	0.2173	0.1577
	Present HSDT results	0.41593	0.3768	0.2938	0.2107	0.1446
**8**	Ref. [53]	0.2358	0.2230	0.1918	0.1555	0.1229
	Present FSDT results	0.2378	0.2255	0.1933	0.1562	0.1231
	Present HSDT results	0.2352	0.2223	0.1909	0.1545	0.1218
**10**	Ref. [53]	0.1509	0.1455	0.1316	0.1134	0.0951
	Present FSDT results	0.1532	0.1476	0.1332	0.1145	0.0957
	Present HSDT results	0.1509	0.1455	0.1314	0.1132	0.0947

**Table 3:** Comparison of the dimensionless buckling load gained by considering various functions of HSDT for a circular nanoplate under both simply supported and clamped boundary conditions.

Condition	Function	*μ* = 0	*μ* = 1	*μ* = 2	*μ* = 3	*μ* = 4
**C**	g_1_(z)	0.01296	0.00720	0.00485	0.00400	0.00355
	g_2_(z)	0.01295	0.00709	0.00480	0.00396	0.00353
	g_3_(z)	0.01296	0.00717	0.00484	0.00399	0.00355
	g_4_(z)	0.01297	0.00722	0.00486	0.00400	0.00356
	g_5_(z)	0.01292	0.00706	0.00478	0.00395	0.00352
**S**	g_1_(z)	0.01096	0.00775	0.00521	0.00426	0.00377
	g_2_(z)	0.01096	0.00767	0.00517	0.00423	0.00375
	g_3_(z)	0.01096	0.007732	0.00520	0.00426	0.00377
	g_4_(z)	0.01096	0.007763	0.00522	0.00427	0.00377
	g_5_(z)	0.01095	0.00760	0.00513	0.00421	0.00373

**Table 4:** Comparison of the dimensionless thermal buckling parameter $\left(\lambda_{T}\right)$ gained by the present study (HSDT and FSDT) with the reference for the annular nanoplate under clamped boundary conditions.

		$\frac{r_{i}}{r_{o}}=0.2$			$\frac{r_{i}}{r_{o}}=0.4$			$\frac{r_{i}}{r_{o}}=0.6$		
	$\frac{h}{r_{o}}$	Ref. [55]	Present Study (HSDT)	Present Study (FSDT)	Ref. [55]	Present Study (HSDT)	Present Study (FSDT)	Ref. [55]	Present Study (HSDT)	Present Study (FSDT)
S–S	0.05	18.547	18.496	18.515	28.641	28.64	28.642	60.198	60.202	60.198
	0.1	17.725	17.650	17.680	26.921	26.922	26.921	53.251	53.283	53.252
	0.15	16.510	16.427	16.468	24.472	24.482	24.472	44.663	44.755	44.663
	0.2	15.069	14.987	15.035	21.709	21.737	21.709	36.436	36.618	36.436
S–C	0.05	40.717	40.687	40.638	61.167	61.107	61.166	122.304	121.893	122.302
	0.1	37.445	37.428	37.386	53.960	53.889	53.961	95.933	95.160	95.934
	0.15	33.022	33.023	32.979	45.108	45.001	45.108	70.612	68.085	70.613
	0.2	28.337	28.340	28.306	36.689	35.867	36.689	51.606	51.539	51.606
C–S	0.05	27.967	26.9487	27.933	49.956	48.898	49.956	109.571	107.756	109.574
	0.1	25.911	24.356	25.948	44.226	42.689	44.228	86.188	83.332	86.189
	0.15	23.085	21.294	23.133	37.136	35.24	37.138	63.635	60.696	63.636
	0.2	20.033	18.184	20.078	30.349	28.46	30.350	46.674	46.299	46.674

**Table 5:** The differences in percentage errors between the present study with the Ref. [55].

		$\frac{r_{i}}{r_{o}}=0.2$		$\frac{r_{i}}{r_{o}}=0.4$		$\frac{r_{i}}{r_{o}}=0.6$	
	$h/r_{o}$	$E_{1}(\%)$	$E_{2}(\%)$	$E_{1}(\%)$	$E_{2}(\%)$	$E_{1}(\%)$	$E_{2}(\%)$
S–S	0.05	0.2749	0.1725	0.0034	0.0034	0.0066	0
	0.1	0.4231	0.2538	0.0037	0	0.0600	0.0018
	0.15	0.5027	0.2543	0.0408	0	0.2059	0
	0.2	0.5441	0.2256	0.1289	0	0.4995	0
S–C	0.05	0.0736	0.1940	0.0980	0.0016	0.3360	0.0016
	0.1	0.0453	0.1575	0.1315	0.0018	0.8057	0.0010
	0.15	0.0030	0.1302	0.2372	0	3.5787	0.0014
	0.2	0.0105	0.1093	2.2404	0	0.1298	0
C–S	0.05	3.6410	0.1215	2.1178	0	1.6564	0.0027
	0.1	6.0013	0.1427	3.4753	0.0045	3.3136	0.0011
	0.15	7.7582	0.2079	5.1055	0.0053	4.6185	0.0015
	0.2	9.2297	0.2246	6.2242	0.0032	0.8034	0

## Data Availability

Data sharing is unavailable due to privacy or ethical restrictions.

## Appendix A

As an example, in this part, the discrete forms of the dimensionless equations of stability for circular nanoplate on the Winkler elastic foundation considering FSDT using DQM are considered. The obtained FSDT stability equations in terms of displacements are as follows:

(A1) $$\begin{array}{l} \frac{1}{2(r^{*}(1-\nu_{12}\nu_{21}))}(2r^{*}(\frac{du_{0}^{*}(r)}{dr^{*}}\delta +r^{*}(\frac{dw^{*}(r)}{dr^{*}}{)}^{2}-\nu_{12}\alpha r^{*}(\frac{dw^{*}(r)}{dr^{*}}{)}^{2}-2\alpha u^{*}(r)\delta +\\ 2r^{*}{}^{2}(\frac{d^{2}u_{0}^{*}(r)}{dr^{*}{}^{2}})\delta +2r^{*}{}^{2}(\frac{dw^{*}(r)}{dr^{*}})(\frac{d^{2}w^{*}(r)}{dr^{*}{}^{2}}))=0 \end{array},$$

(A2) $$\begin{array}{l} \frac{1}{12}\frac{1}{r^{*}\delta (1-\nu_{12}\nu_{21})}(\delta^{2}(\frac{d^{2}\varphi^{*}(r)}{dr^{*2}})r^{*2}+\delta^{2}(\frac{d\varphi^{*}(r)}{dr^{*}})r^{*}-\alpha \delta^{2}\varphi^{*}(r)\\ +12r^{*2}K_{s}\beta \varphi^{*}(r)\nu_{12}\nu_{21}-12r^{*2}K_{s}\beta \varphi^{*}(r)+12r^{*2}K_{s}\beta (\frac{dw^{*}(r)}{dr^{*}})\nu_{12}\nu_{21}\\ -12r^{*2}K_{s}\beta (\frac{dw^{*}(r)}{dr^{*}})=0 \end{array},$$

(A3) $$\begin{array}{l} \frac{1}{r^{*3}\delta }(-K_{s}\beta r^{*3}\delta (\frac{d\varphi^{*}(r)}{dr^{*}})-K_{s}\beta r^{*3}\delta (\frac{d^{2}w^{*}(r)}{dr^{*2}})-K_{s}\beta r^{*2}\delta \varphi^{*}(r)-K_{s}\beta r^{*2}\delta (\frac{dw^{*}(r)}{dr^{*}})+\\ N^{*}r^{*3}\delta (\frac{d^{2}w^{*}(r)}{dr^{*2}})+N^{*}r^{*2}\delta (\frac{dw^{*}(r)}{dr^{*}})+K_{w}^{*}w^{*}(r)r^{*3}-\mu^{*}N^{*}\delta (\frac{d^{4}w^{*}(r)}{dr^{*4}})r^{*3}-\\ \mu^{*}N^{*}\delta (\frac{dw^{*}(r)}{dr^{*}})+\mu^{*}N^{*}\delta (\frac{d^{2}w^{*}(r)}{dr^{*2}})-2\mu^{*}N^{*}\delta (\frac{d^{3}w^{*}(r)}{dr^{*3}})r^{*2}-\mu^{*}K_{w}^{*}(\frac{d^{2}w(r)}{dr^{*2}})r^{*3}\\ -\mu^{*}K_{w}^{*}(\frac{dw^{*}(r)}{dr^{*}})r^{*2})=0 \end{array}.$$

Applying DQM, the discrete forms of Equations (A1)–(A3) are as follows:

(A4) $$\begin{array}{l} \frac{\delta }{\left(1-\nu_{12}\nu_{21}\right)}\sum_{j=1}^{9}A_{ij}^{\left(1\right)}u_{0}^{*}(r)+\frac{\delta }{2(1-\nu_{12}\nu_{21})}\sum_{j=1}^{9}A_{ij}^{\left(1\right)}w_{0}^{*}(r_{j})\sum_{j=1}^{9}A_{ij}^{\left(1\right)}w_{0}^{*}(r_{j})-\\ \frac{\delta \nu_{12}\alpha }{\left(1-\nu_{12}\nu_{21}\right)}\sum_{j=1}^{9}A_{ij}^{\left(1\right)}w_{0}^{*}(r_{j})\sum_{j=1}^{9}A_{ij}^{\left(1\right)}w_{0}^{*}(r_{j})-\frac{\delta \alpha }{\left(1-\nu_{12}\nu_{21}\right)}\sum_{j=1}^{9}w_{0}^{*}(r_{j})+\\ \frac{\delta }{\left(1-\nu_{12}\nu_{21}\right)}\sum_{j=1}^{9}r_{j}^{*}A_{ij}^{\left(2\right)}u_{0}^{*}(r)+\frac{\delta }{\left(1-\nu_{12}\nu_{21}\right)}\sum_{j=1}^{9}r_{j}^{*}A_{ij}^{\left(2\right)}w_{0}^{*}(r_{j})\sum_{j=1}^{9}A_{ij}^{\left(1\right)}w_{0}^{*}(r_{j})=0 \end{array},$$

(A5) $$\begin{array}{l} \frac{1}{12}(\frac{\sum_{j=1}^{9}r_{j}^{*}C_{ij}^{\left(2\right)}\varphi^{*}(r_{j})}{\left(1-\nu_{12}\nu_{21}\right)}+\frac{\nu_{12}\alpha \sum_{j=1}^{9}A_{ij}^{\left(1\right)}\varphi^{*}(r_{j})}{\left(1-\nu_{12}\nu_{21}\right)}-\frac{\nu_{12}\alpha \sum_{j=1}^{9}\frac{1}{r_{j}^{*}}\varphi^{*}(r_{j})}{\left(1-\nu_{12}\nu_{21}\right)})\delta +\frac{1}{12}(\frac{\sum_{j=1}^{9}A_{ij}^{\left(1\right)}w^{*}(r_{j})}{\left(1-\nu_{12}\nu_{21}\right)}\\ +\frac{\nu_{12}\alpha \sum_{j=1}^{9}\frac{1}{r_{j}^{*}}\varphi^{*}(r_{j})}{\left(1-\nu_{12}\nu_{21}\right)})\delta -\frac{1}{12}(\frac{\nu_{12}\alpha (\sum_{j=1}^{9}A_{ij}^{\left(1\right)}\varphi^{*}(r_{j}))}{\left(1-\nu_{12}\nu_{21}\right)}+\frac{\alpha \sum_{j=1}^{9}\frac{1}{r_{j}^{*}}\varphi^{*}(r_{j})}{\left(1-\nu_{12}\nu_{21}\right)})\delta \\ -\frac{K_{s}\beta (\sum_{j=1}^{9}r_{j}^{*}\varphi^{*}(r_{j})+\sum_{j=1}^{9}A_{ij}^{\left(1\right)}w^{*}(r_{j}))}{\delta }=0 \end{array},$$

(A6) $$\begin{array}{l} K_{s}\beta (\sum_{j=1}^{9}A_{ij}^{\left(1\right)}\varphi^{*}(r_{j})+\sum_{j=1}^{9}A_{ij}^{\left(2\right)}w_{0}^{*}(r_{j}))+K_{s}\beta (\sum_{j=1}^{9}\varphi^{*}(r_{j})+\sum_{j=1}^{9}\frac{1}{r_{j}^{*}}A_{ij}^{\left(1\right)}w_{0}^{*}(r_{j}))-\\ N^{*}(\sum_{j=1}^{9}A_{ij}^{\left(2\right)}w_{0}^{*}(r_{j})+\sum_{j=1}^{9}\frac{1}{r_{j}^{*}}A_{ij}^{\left(1\right)}w_{0}^{*}(r_{j}))-\frac{K_{w}^{*}\sum_{j=1}^{9}w_{0}^{*}(r_{j})}{\delta }-\mu^{*}(-N^{*}(\sum_{j=1}^{9}A_{ij}^{\left(4\right)}w_{0}^{*}(r_{j})+\\ 2(\sum_{j=1}^{9}\frac{1}{r_{j}^{*3}}A_{ij}^{\left(1\right)}w_{0}^{*}(r_{j}))-2(\sum_{j=1}^{9}\frac{1}{r_{j}^{*2}}A_{ij}^{\left(2\right)}w_{0}^{*}(r_{j}))+\sum_{j=1}^{9}\frac{1}{r^{*}}A_{ij}^{\left(3\right)}w_{0}^{*}(r_{j}))-\\ \frac{1}{\delta }K_{w}^{*}(\sum_{j=1}^{9}A_{ij}^{\left(2\right)}w_{0}^{*}(r_{j}))-N^{*}(\sum_{j=1}^{9}\frac{1}{r_{j}^{*}}A_{ij}^{\left(3\right)}w_{0}^{*}(r_{j})+\sum_{j=1}^{9}\frac{1}{r^{*3}}A_{ij}^{\left(1\right)}w_{0}^{*}(r_{j})-\sum_{j=1}^{9}\frac{1}{r^{*2}}A_{ij}^{\left(2\right)}w_{0}^{*}(r_{j})+\\ \frac{1}{\delta }K_{w}^{*}(\sum_{j=1}^{9}\frac{1}{r^{*}}A_{ij}^{\left(1\right)}w_{0}^{*}(r_{j}))=0 \end{array}$$

where $\alpha =\frac{E_{2}}{E_{1}};\beta =\frac{G_{13}}{E_{1}}$ are considered; also, $K_{s}=5/6$.
